# Mixture of experts extra tree-based sEMG hand gesture recognition

**DOI:** 10.1038/s41598-026-40305-z

**Published:** 2026-03-02

**Authors:** Naveen Gehlot, Ashutosh Jena, Rajesh Kumar, Surender Hans, Mahipal Bukya, Pilani Nkomozepi

**Affiliations:** 1 Manipal Institute of Technology, Manipal Academy of Higher Education, Manipal, India; 2 Department of Electrical Engineering, Malaviya National Institute of Technology Jaipur, Jaipur, Rajasthan India; 3 Department of Human Anatomy and Physiology, Faculty of Health Sciences, University of Johannesburg, 2094 Johannesburg, South Africa; 4 Department of Electrical and Electronics Engineering, Manipal Institute of Technology Bengaluru, Manipal Academy of Higher Education, Manipal, India

**Keywords:** Artificial intelligence, Mixture of experts, Extra tree, Machine learning classifier, Hand gesture recognition, Electromyography, Computational biology and bioinformatics, Engineering, Health care, Mathematics and computing

## Abstract

Human hand gesture recognition has emerged as a significant research interest in robotic hand control using surface electromyography. It is popular due to its non-invasive nature and ability to capture gesture movements. However, proper handling of overfitting and biases in gesture classification with multiple gestures is a common issue in the development of a generalized classifier. To address this issue, a Mixture of Experts Extra Tree classifier proposes to identify hand gestures. This model utilizes the concept of a Mixture of Experts, which combines individual highly accurate machine learning models (Extra Tree), referred to as experts, each focusing on a specific class, and a fully trained model for all classes, known as the gate (Extra Tree model), is employed to weigh the output of individual expert models. In this study, four subjects performing six hand gesture movements were collected, and their identification was evaluated among eleven models, including the Mixture of Experts Extra Tree classifier. The model was also evaluated on a publicly available dataset containing fifteen gesture classes. Results demonstrate that the Mixture of Experts Extra Tree classifier outperformed the other algorithms considered in this study, accurately identifying hand gesture movements. The MEET classifier achieved accuracies of 86.8%, 89.2%, 87.9%, and 78.4% for four subjects on collected data, and improved mean accuracy by 1.25% on the public dataset.

## Introduction

The promotion of technology has advanced from manually operated devices to intelligently controlled devices over the past few years. In the coming years, industries are seeking a more comfortable form of control, which has led to the recent growth of research on Human-Computer Interaction (HCI)-based controllability. While the study on HCI has the potential to revolutionize various industrial sectors, ranging from healthcare to the military, the main concern of this study revolves around HCI in achieving controllability in prostheses for hand amputees. HCI provides a platform for interaction between humans and machines to achieve common objectives. This interaction can be achieved using various modalities, including speech signals, image signals, sound, thermal readings, other bodily features, and biopotential signals^1,2^.

Similarly, human activity recognition approaches exploit video or skeleton-based features for HCI applications, providing complementary methods for gesture and activity recognition. The preferred form of interaction for a hand amputee to control prostheses is a biopotential signal, which can be either a brain signal (EEG) or a muscle signal (EMG)^3^. However, EMG signals have recently gained significant interest in achieving prosthesis control. The main reason is the proximity of the acquisition device to the acting muscle and the reduced influence of noise from other body parts influencing the original signal. EMG is a method for capturing and displaying biopotential signals generated by muscles, yielding a time-dependent signal as output. These signals can be acquired using either needle electrodes (iEMG) or surface electrodes (sEMG). The majority opt for non-invasive assessments, such as sEMG. Typically, Ag/AgCl-based electrodes are employed, along with conductive gel, to reduce the impedance of the skin surface^4,5^.

The complete framework to develop prostheses for hand amputees generally involves three stages. The first and last stages involve the acquisition and actuation on the hardware level. The second stage involves processing. This study mainly focuses on the second stage. This stage deals with processing the acquired sEMG signal and producing a categorical output for subsequent stages. This stage consists of two main parts. The first part involves data preprocessing. The second part applies a model to predict categorical outputs based on the preprocessed data.

The first part, preprocessing, is essential to remove the noise in acquired data. These noises aid in the misclassification of the signal. The input to a machine learning classifier is a numerical feature representing a window of signal. Noise across different signals has similar properties and, hence, similar numerical features. When noise is dominant, the features corresponding to it become dominant. The features computed over a noisy signal are the sum of the signal feature and the noise feature. The signal feature becomes redundant when the noise feature is higher; training on such data compromises the objective of gesture classification and instead classifies the noise. This is a training error and must be avoided prior to classifier training. When such erroneous classifiers are deployed, they may cause improper actuation of prosthetics and other biomechanics, which may result in invalid diagnoses during rehabilitation^6,7^. Hence, we preprocess the raw signal to enhance model reliability. The noises in a typical sEMG signal include user variations and contamination from sources such as motion artifacts (due to the electrode interface and electrode cable), power line interference, ambient noise, and inherent noise due to electrical and electronic equipment^8^. As mentioned earlier, classification output depends upon the dominance of noise. A bandpass filter is applied to preserve the actual sEMG signal by removing common noise, and a notch filter is used to eliminate power line interference noise (50 Hz)^9^. The second part involves machine learning classifiers (MLCs) and deep learning classifiers (DLCs) for categorical output prediction. Deep learning architectures are most commonly used due to their strong performance in various fields, such as image classification and object detection, where large datasets are available^10^. This deep learning architecture includes artificial neural networks^11^, convolutional neural networks (CNNs)^12^, long short term memory (LSTM) networks^13^, graph-based networks^14^, and other hybrid combinations of these networks^15^.

Similarly, hand gesture recognition (HGR) can be achieved using MLCs or DLCs. The most applied DLC in this regard is a CNN. Atzori et al. demonstrated the reliability and high accuracy of a simple CNN in an HGR dataset that includes the data of both amputee and non-amputee human subjects in ^16^. As the dataset becomes larger, the depth of the CNN has to be reconsidered. Although it may seem like a simple task, transfer learning can aid in reducing the burden of this task^17^. Côté-Allard U. et al. demonstrated the applicability of transfer learning in a large dataset in^18^. Additionally, they also demonstrated that their method is invulnerable to the effect of muscle fatigue. The DLCs do not require extracted features; rather, they have inherent feature extractability. However, sometimes, to improve the HGR accuracy, features are extracted from the signal, and then DLCs are used for HGR. In^19^, Geng et al. demonstrated that high-density EMG signals could be converted into instantaneous sEMG images, and a CNN network can be coupled to provide better accuracy. Similarly, in^20^, Huang et al. utilized the spectrogram of the sEMG signal as an image feature for a CNN and an LSTM network to link the spatial features in time. Converting the sEMG signal to an image and applying a CNN can be costly when processing time is of the essence. In such cases, to reduce preprocessing time, an sEMG signal can be preprocessed in real-time using moving feature computational techniques, and then this real-time, preprocessed signal can be input into a recurrent neural network. Such a case is demonstrated in^21^, where a moving average filter is used to obtain the real-time preprocessed signal along with an LSTM network.

Despite the very high performance of DLCs, they consume a large storage space in the hardware and a considerable amount of time to process inputs to provide outputs. A good preprocessing technique may help partially by reducing the large processing time^22^. However, the problem of large storage space is unavoidable. To overcome this problem, machine learning is preferred in low-cost, real-time HGR with prostheses. Pérez et al. reviewed the application of support vector machine (SVM) in EMG-based HGR in^23^. In their article, it is concluded that a radial basis function kernel followed by principal component analysis is predominantly considered while tuning SVM for better accuracy. Sara Abbaspour et al.^24^ classified hand gestures using linear discriminant analysis (LDA), k-nearest neighbors (KNN), decision tree (DT), SVM, multi-layer perceptron (MLP), etc., and compared them based on accuracy and performance to conclude MLP to have the best of both. In their study, they also eliminated a few features to improve accuracy and performance. A similar analysis on accuracy is done by Smita Bhagwat et al. in^25^, where they applied KNN, SVM, and quadratic discriminant analysis for HGR.

In order to match an accuracy comparable to that obtained with DLCs, the selection of refined feature extraction techniques and MLC models is still an open area of research. Research in the refinement of MLC models suggests that combinational MLC models, such as ensemble MLCs, show superior performance as compared to the standalone MLCs. In^26^, Ji-Won Lee et al. applied four MLCs that include SVM, KNN, Naive Bayes (NB), and DT for HGR, and in addition, they also mentioned the performance of Ensemble with their dataset. However, these models are considered individually, which may show biasing when two or more classes have very similar features. Binish Fatimah et al.^27^ applied ensemble bagged trees and ensemble subspace discriminant in addition to SVM and KNN for HGR and obtained a mean accuracy of 99.5% and 93.5% on the UCI and NinaPro DB5 datasets, respectively.

A substantial body of literature has explored DLCs and ensemble-based MLCs for sEMG-based HGR. Most existing approaches rely either on standalone classifiers or on ensemble models trained using the complete multi-class dataset, which can sometimes restrict their flexibility and generalization across various gestures. In contrast, a combinational learning strategy known as the Mixture of Experts (MoE) has been introduced to address limitations associated with multi-class learning. The key strength of the MoE lies in its ability to combine multiple expert-based models, each trained on a specific subset of class data, together with a gating network that controls their contributions. Since the expert models and the gating network give independent decisions, their fusion helps reduce overall model biasing, leading to improved classification performance^28,29^.

Additionally, MLCs trained on datasets with fewer class labels often learn more precise decision boundaries compared to those trained on highly multi-labeled datasets. However, effective implementation of MoE requires careful tuning of both the expert models and the gating mechanism. Motivated by these observations, this study integrates the MoE framework with an ensemble learning approach by employing Extra Trees (ET) as expert classifiers, resulting in a framework referred to as the Mixture of Experts Extra Trees (MEET), proposed to enhance classification performance in sEMG-based HGR.

The need for HCI-based control of prosthetic devices motivates this study. In this context, sEMG is widely regarded in the literature as a preferred technique for gesture recognition. A key component of this recognition process is categorizing different gestures using intelligent control techniques for real-time control. Given the constraints of limited data and the use of low-end processing units, machine-learning models are often preferred. However, traditional machine learning classifiers face limitations, such as handling multiple gestures and addressing biases. These challenges are addressed by the proposed model in this study, known as the Mixture of Experts Extra Tree classifier.

The major contributions of this study can be summarized as follows:

1. The study employs handcrafted feature extraction, which encompasses seventeen features derived from the signal acquired using a two-channel Biopac MP150 acquisition device.
2. For HGR, a Mixture of Experts Extra Trees (MEET) model is proposed.
3. The proposed method is compared with ten machine learning models and evaluated on the basis of accuracy, precision, recall, and F1-score.

The article consists of four sections; Section II elaborates on the methodology, which includes acquisition, pre-processing, and the functioning of MEET. In Section III, results have been presented and briefly discussed. Section IV covers the conclusion and future scope of this methodology.

**Fig. 1:**
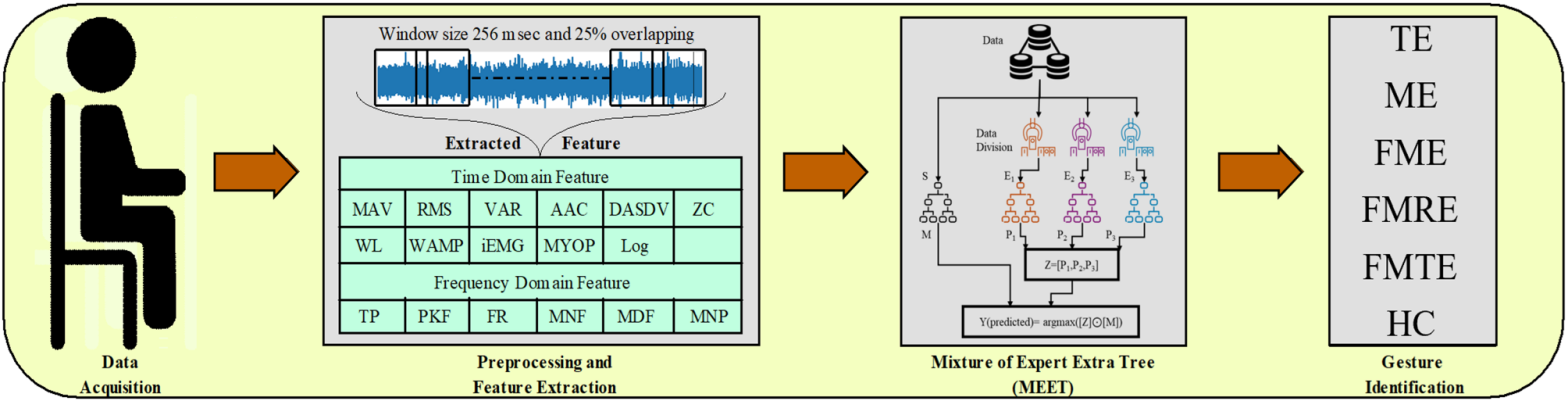
Workflow of MEET-based HGR.

## Materials and methods

The study is executed in four independent stages as illustrated in Fig. 1. Stage I comprises data acquisition. Stage II comprises pre-processing and feature extraction. Stage III comprises HGR. Stage IV comprises performance evaluation.

### Data acquisition

The physical data from the environment is converted into values through measuring or sensing devices; this process is called data acquisition. Based on the source of data, this can be classified into two types: primary and secondary. Secondary data is data derived from another study directly or after processing, whereas primary data is a direct collection of data from the physical environment. In this study, both kinds have been used. Initially, the proposed MLC is evaluated on data acquired using BIOPAC MP150. To demonstrate the robustness of the model over widely used sEMG data, it is tested on secondary data obtained from publicly available repositories titled “Individual and Combined Fingers Movements”^30,31^. This data has been acquired from arm muscles using 8 channels. It covers the 15 most frequently used gestures, which makes it a good choice for proposed model testing. The former approach, which involves data acquisition with BIOPAC MP150, is detailed below, and more about the public dataset used for testing is detailed later in Section Evaluation with Publicly Dataset.

Initially, for data acquisition participants are provided a scripted and verbal explanation of the task they are required to perform. The willingness of participants is recorded in a consent form, where they acknowledge the need to share their data for research purposes. Four healthy adult male and female subjects participated in this study. The participants were instructed to perform activities in a predetermined order, with six actions being examined. Each of these exercises is executed in three steps. The participants are advised to maintain and restrict their movements during recording^32^.

Stage I: at this stage, participants are briefed on the exercise two minutes before commencing the assignment and advised to relax.

Stage II: at this stage, participants are instructed to perform the task continuously for 40 seconds at an approximate interval of 1–2 s.

Stage III: participants are given time to recuperate after each activity. Muscle signals may deteriorate as the muscles become fatigued.

The EMG signal is acquired using AcqKnowledge software version 4.4, which is paired with the BIOPAC MP150 controller for signal acquisition. The hardware arrangement for data acquisition, which involves placing disposable Ag/Ag-Cl surface electrodes on the participant’s forearm muscle to capture the train of muscle-activated potentials from the extensor digitorum and flexor pollicis longus muscles^33–35^, which makes the two-channel sEMG signal. Shielded cable electrodes are used to acquire data from the Ag/AgCl skin interface electrode. A TEL-100MC filter amplifier with a gain of 1000 is used to bring the low-voltage signal to a controller-readable voltage signal. The data is captured at a rate of 2000 Hz to ensure the acquisition of the entire bandwidth of the EMG signal, which varies between 10 and 500 Hz.

### Preprocessing and feature extraction

**Fig. 2:**
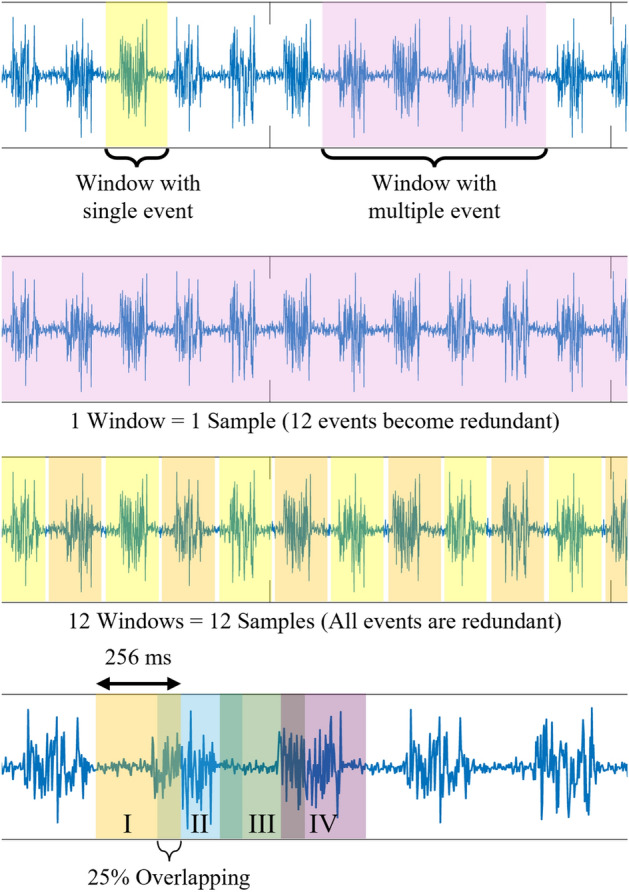
Demonstrating the need of windowing in pre-processing of sEMG signals. Top: specification of single and multiple events. Middle: the second and third pictures from the top indicate the relevance of the correct windowing scheme to avoid redundancy during windowing. Bottom: overlapping windowing technique.

During acquisition, the physical EMG signal is corrupted with several noises at different stages before it reaches the controller. These noises can have undesirable effects on the final outcomes of HGR. To ensure the quality of the outcome, the noises are removed using common filtering techniques, which are designed to be suitable to a particular type of noise.

The low-frequency noise in the signal due to the interference of the other physiological signals in the proximity of the electrode surface and the other high-frequency noise produced during analog-to-digital conversion is eliminated using a band-pass filter, which allows only the range of the EMG signal (i.e., 10–500 Hz), eliminating the rest. Additionally, a notch filter with a cutoff frequency of 50 Hz is used to eliminate the power line interference due to the electrical appliances in the proximity. The transfer function of a bandpass filter is as given below:

1 $$\begin{aligned} F(x) = H\frac{x^4}{x^4+a_3x^3+a_2x^2+a_1x+a_0} \end{aligned}$$

Here, *H* is an indication of amplification, and $a_n$ denotes the coefficients of frequency components of the signal that are absent in the output of filter^36^.

Post filtering, the signal is sent for feature extraction, which is a crucial step in deciding the efficacy of machine-learning models. Features are numerical representations of a signal. The signal of length *l* and width *b* having *f* features (in this case, channels) may be represented as $n\times f$ numerical values, where *n* is the number of features computed. However, obtaining a single sample of features from an entire signal may include all the repetitions of the same activity. This way, all the different events of the same activity may become redundant (refer to Fig. 2). Therefore, rather than computing a single sample of features from the entire signal, multiple samples of features are computed in intervals of the same signal, which is referred to as windowing. Normally, a window should include the complete event of an activity for proper feature extraction; however, to avoid a potential delay in hardware actuation, a window of 256 ms is preferred. When such a length of signal is considered, the current event of an activity may not be completely enclosed within that window, and some part of the same event may be present in the next window. Moreover, these windows are later used to compute features. When these windows are converted into features, the temporal dependency of two consecutive windows is missed. Therefore, in this context, an overlapping window is preferable to take into consideration the time dependency of consecutive windows in the extracted features (refer to Fig. 2). Overall, windowing is similar to segmenting signals consecutively in equal intervals. Since the potential values of the sEMG signal are related in the time domain, they have to be reflected in the extracted features. To achieve this, consecutive windows are overlapped such that the preceding window also shares information from the past window, simulating the time dependency of potential values of the sEMG signal. This mode of feature extraction is referred to as the overlapping window feature extraction. In this study, a window length of 256 ms and an overlap of 25% are considered between successive windows^37^.

A single window may be represented in the form of several features; however, most of such features may not be accountable for HGR. In this study, a set of seventeen essential features for HGR is considered from^32,38^. These features include Mean Absolute Value, Variance, Difference Absolute Standard Deviation Value, Waveform Length, Integrated Electromyogram, Log Detector, Root Mean Square, Average Amplitude Change, Zero Crossing, Willson Amplitude, and Myopulse, which are time domain features, and total power, frequency ratio, median frequency, peak frequency, mean frequency, and mean power as frequency domain features. These features are extracted from each window of the signal. Finally, the number of windows decides the number of samples in the dataset for MLC training and testing.

### Hand gesture recognition (HGR)

The final stage is HGR, where the handcrafted features are provided as inputs to the proposed model MEET, which comes into the picture. Apart from the proposed model, this study employs ten MLCs for a comparative analysis; these MLCs are listed in Table 1. In this section, the proposed model has been elaborated. In addition, various performance metrics utilized during comparative analysis are briefly described.

**Table 1:** MLCs utilized for comparative analysis.

MLC	Reference	Parameters	Value	MLC	Reference	Parameters	Value
DT	^39^	Criterion	Gini	LR	^40^	Penalty	l2
		Splitter	Best			Tol	0.0001
		min_samples_split	2			C	1
		min_samples_leaf	1			intercept_scaling	1
RF	^41^	n_estimators	100			Solver	lbfgs
		Criterion	Gini			max_iter	100
		min_samples_split	2	NB	^40^	Alpha	1
		min_samples_leaf	1			force_alpha	True
		max_features	Sqrt			fit_prior	True
GB	^42^	learning_rate	1	KNN	^43^	n_neighbors	5
		n_estimators	100			leaf_size	30
		Criterion	friedman_mse			p	2
		min_samples_split	2			Metric	Minkowski
		min_samples_leaf	1	ET	^44^	n_estimators	100
		max_depth	3			Criterion	Gini
		Tol	0.0001			min_samples_split	2
ADB	^45^	n_estimators	50			min_samples_leaf	1
		learning_rate	1			max_features	Sqrt
SVM	^46^	C	1	MEET	Proposed	Gating network	ET
		Kernel	rbf			Experts	ET
		Degree	3			n_estimators	100
		Gamma	Scale			Criterion	Gini
		decision_function_shape	ovr			min_samples_split	2
		cache_size	200			min_samples_leaf	1
BAG	^47^	n_estimators	10			max_features	Sqrt
		max_samples	1				
		max_features	1				
		Bootstrap	True				

*DT* decision tree, *RF* random forest, *GB* gradient boost, *ADB* adaboost, *SVM* support vector machine, *BAG* bagging, *LR* logistic regression, *NB* naive bayes, *KNN* K nearest neighbor, *ET* extra tree, *MEET* mixture of expert extra tree.

The proposed model (MEET) is the combination of the concept of the Mixture of Experts (MoE) and the MLC Extra Tree (ET). In our previous studies^32,48^, it has been demonstrated that the ET model for HGR provides a very high accuracy. Therefore, ET has been chosen as the expert. The concepts driving MoE and its extension (MEET) are described below.

Background of mixture of experts (MoE): the key idea of the mixture of experts (MoE) model is introduced in^28,29^. The main principle behind it is “divide and conquer”, which is common in the field of computational algorithms.

**Fig. 3:**
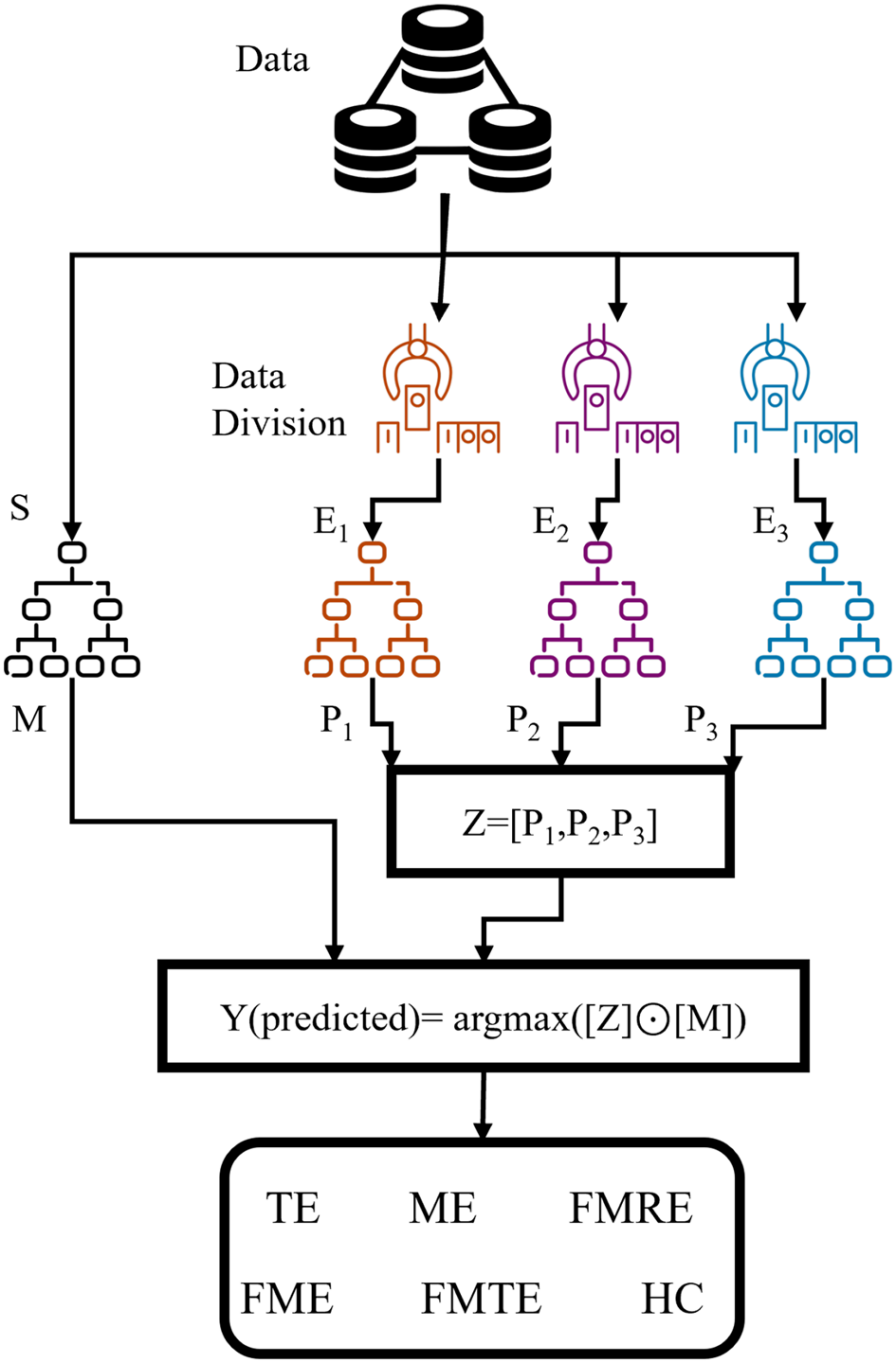
MEET: mixture of experts extra tree architecture.

According to this principle, by dividing a problem into several smaller problems, the complexity of the original problem can be reduced. The MoE consists of a set of experts and a gating network that combines the decisions from these experts. Utilizing the principle of divide and conquer, the dataset is first divided into smaller subsets on which an expert is trained. This is combined with the gating network to obtain the final model as illustrated in Fig. 3.

Proposed mixture of experts extra tree (MEET):

The proposed MEET framework is built around a class-specific expert learning strategy combined with a gated decision integration process. Rather than training all classifiers on the complete multi-class dataset, MEET decomposes the overall classification task into smaller, more focused learning tasks. Each expert Extra Tree classifier is trained using data corresponding only to a selected group of gesture classes, allowing it to learn more distinct decision boundaries. Alongside these experts, an Extra Tree–based gating model is trained on the full dataset to adaptively weight the outputs of individual experts for final decision-making. This architectural configuration reduces classification bias and improves generalization across gestures when compared to traditional Mixture of Experts models.

To place the effect of bias in a multi-class setting into perspective, it is useful to consider that the least accuracy that may be obtained with any biased classifier is $\frac{1}{n_{class}}\times 100\%$, where $n_{class}$ is the number of classes. Therefore, in the case of a multi-class problem, for effective training, the idea is to avoid bias in the model. To achieve this, a gating network is trained on the same input data in addition to the MLCs because decisions from two different models reduce the overall bias in the outcome. This will become clear when the working of MEET is discussed.

Furthermore, the classifier’s mixture structure allows for independent training of sibling modules in parallel, reducing total training time and improving accuracy. The sibling classifiers contain fewer parameters and are less prone to overfitting, allowing for high accuracy with minimum hyperparameter modification. The proposed MEET is depicted in Fig. 3, while its algorithmic formulation is illustrated in Algorithm 1.

The minimum number of classifiers required is determined based on the following equation.

2 $$\begin{aligned} n_{MLC}= \Big \lceil \frac{N}{a} \Big \rceil +1 \end{aligned}$$

Where *N* represents the number of classes in the data sample, *K* is the number of channels, *p* is the number of extracted features, and *a* is a variable that may vary based on the data class assigned to each expert classifier. In the data sample acquired for this study, *a* is set to 2.

The $n_{MLC}$ is an integer rather than a fraction. In the equation, the additional 1 signifies the classifier that assigns weighted probabilities to all the base classifiers and chooses the most accurate output based on the probability multiplier.

The operational framework of the MEET architecture is illustrated in Fig. 3. Initially, the number of the network’s classifiers is determined in accordance with Eq. 2. This study involves 6 distinct classes; therefore, the required number of MLCs is 4. In this, 3 are expert classifiers, each adept for two classes, and 1 gate classifier adept to the entire data. The gating network is responsible for adjusting the weights attributed to the outputs of the expert classifiers. Therefore, the final decision is the combined decision of the gating network and one of the experts, which justifies the reduction of bias. The output depends upon the maximum value of the expert’s output product and the weightage it carries, which is decided by the gating network. The distribution of data to the experts is elaborated in the next paragraph.

The data undergoes an initial segmentation phase, meticulously partitioned by class assignment to each expert classifier. This segmentation ensures that each expert classifier trains on data relevant to its designated classes, thereby avoiding overlap or confusion during the training process. For instance, Expert $E_1$ is entrusted with the training data corresponding to classes 1 and 2, Expert $E_2$ handles classes 3 and 4, and Expert $E_3$ manages classes 5 and 6. Simultaneously, gate classifier *S* is trained using the entire class data, facilitating its role in determining the final output.

Ultimately, the output of the MEET architecture is a composite of the individual expert classifier outputs—$P_1$ for $E_1$, $P_2$ for $E_2$, and $P_3$ for $E_3$—alongside the weighted output, denoted as M, generated by gate classifier *S*. This output is mathematically formalized based on the Hadamard product of stacked output of experts denoted as *Z* and gate classifier outputs *M* to yield the MEET architecture’s final prediction or decision output shown in Eq. (3).

3 $$\begin{aligned} Y(predicted) = \arg \max \left( [Z]\odot [M] \right) \end{aligned}$$

**Algorithm 1:**
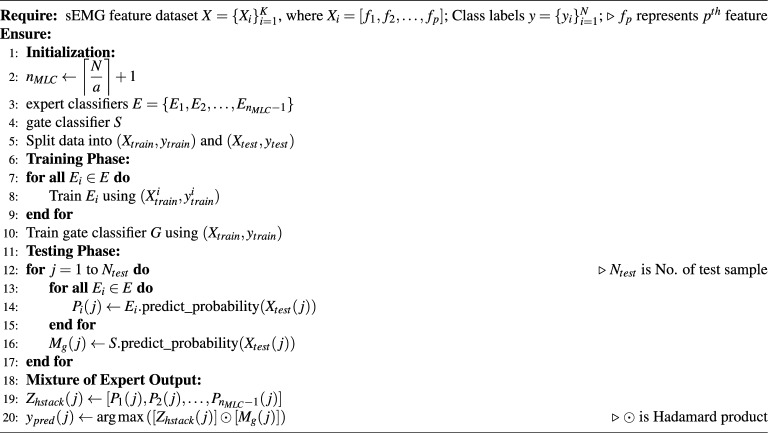
MEET algorithm.

## Results and discussion

To validate the proposed model, it was applied to datasets from four individuals, each containing 480,000 raw samples, resulting in a total of 1,920,000 samples collected. These datasets are collected by performing six gestures: TE, ME, FME, FMTE, FMRE, and HC. These datasets are then applied to the proposed model, MEET, and compared against existing models, including DT, RF, GB, ADB, SVM, BAG, LR, NB, KNN, and ET. All the simulations are conducted using Python 3.11.3, with machine learning classifiers implemented using the Scikit-learn library version 1.2.2^49^, utilizing the default parameter values set by the library. All these models are trained on 70% of the total data and validated using the remaining 30% of the data.

The model is evaluated based on performance metrics discussed in Table 2, including accuracy, precision, recall, and F1-score. Accuracy signifies the overall correct prediction made by the model. Precision indicates the proportion of true positive predictions among all positive predictions made by the model. Recall metrics quantify all positive instances captured by the model.

**Fig. 4:**
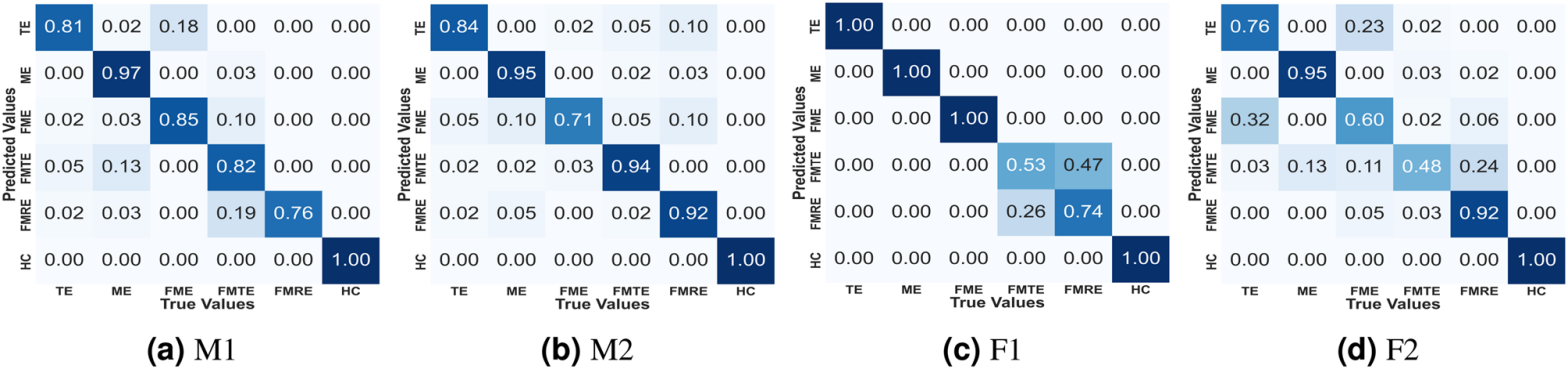
Confusion matrix of MEET.

Another metric, which is also known as the F1-score, is the harmonic mean of the precision and recall. F1-score identifies the model accuracy in a scenario consisting of uneven sample distribution among various classes. These metrics for the model are calculated using the confusion matrix shown in Fig. 4. This matrix indicates the number of correct and incorrect predictions. In Fig. 4, the horizontal axes represent the actual label, and the vertical axes represent the predicted label. The diagonal values indicate the accuracy of an MLC, while the off-diagonal elements indicate the MLC’s error in classification. Consequently, Fig. 4 suggests that the proposed model outperforms the other HGR method in terms of accuracy. Moreover, the generalizability of the above statement is verified by experiments on four different subjects, and the performance of MEET in all those cases is illustrated in Figs. 4a–d. M1, M2, F1, and F2 represent Male-1, Male-2, Female-1, and Female-2, respectively.

**Table 2:** Performance metrics for machine learning classifiers.

Performance Parameter	Formula
$A_{cc}$	$\frac{Tp+Tn}{Tp+Fp+Tn+Fn}$
$P_{re}$	$\frac{Tp}{Tp+Fp}$
$R_{ec}$	$\frac{Tp}{Tp+Fn}$
$$F_s$$	$$\frac{2*Pre*Rc}{Pre+Rc}$$

Here, $A_{cc}$ represents the Accuracy parameter, $P_{re}$ denotes the Precision parameter, $R_{ec}$ stands for the Recall parameter, and $F_s$ represents the F1-score parameter of the performance. Also, *Tp* represents the true positive, *Fp* indicates the false positive, *Tn* represents the true negative, and *Fn* represents the false negative^37^

**Fig. 5:**
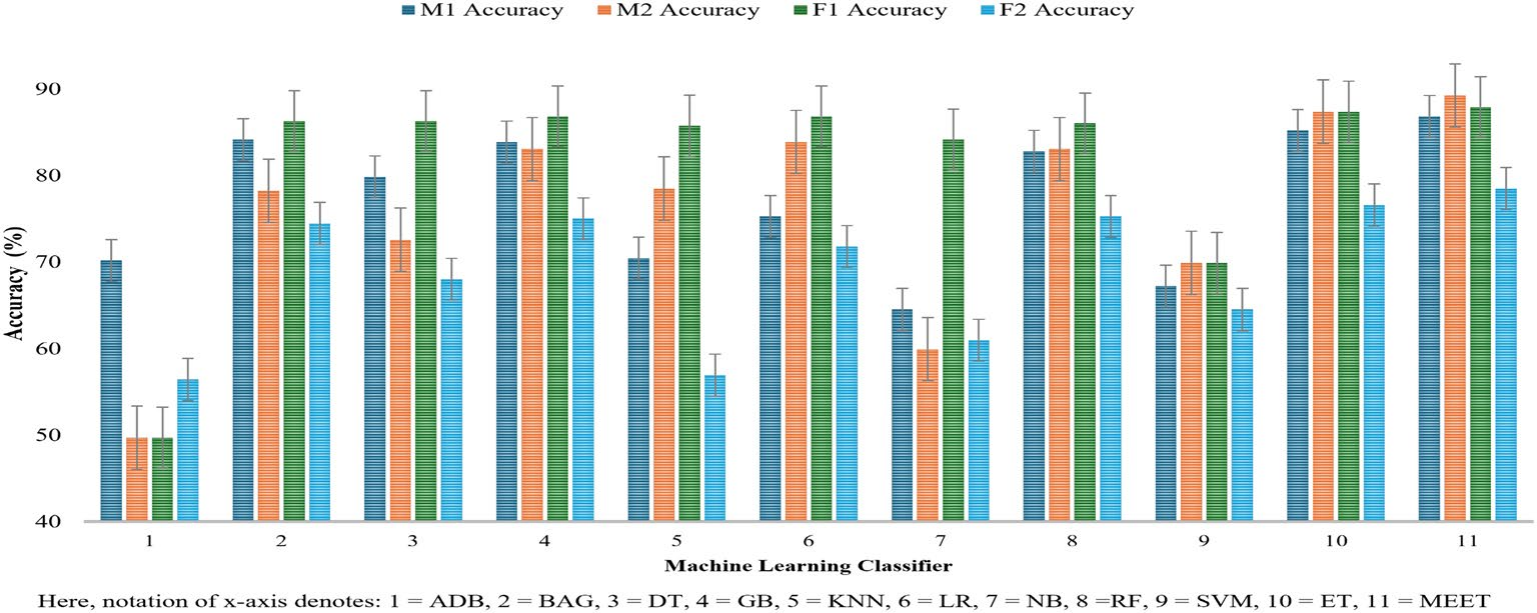
Accuracy plot of all the classifiers with MEET.

Figure 5 demonstrates that the proposed architecture enhances the classification rate. The bars in this figure indicate the percentage of accuracy of an MLC. The group of four bar charts indicates the accuracy for all four participants individually.

This allows visual comparison of the accuracy of every MLC tested with all four subjects. The figure shows that the proposed model has an accuracy of 86.80% for subject M1, 89.20% for subject M2, 87.90% for subject F1, and 78.40% for subject F2. This is very high compared to the other MLCs taken into consideration. The second-highest MLC among all others is ET, which attains 85.20% for subject M1, 87.30% for subject M2, 87.15% for subject F1, and 76.60% for subject F2.

These results reflect the model’s ability to accurately predict the correct gesture among the set of gestures tested. In Figure 5 the best classifier among all subjects is the proposed MEET, and the second best is the ET which is the base model of the proposed MEET. Further positions of models based on accuracy varies with subject, and ADB consistently holds the lowest accuracy across all subjects.

**Fig. 6:**
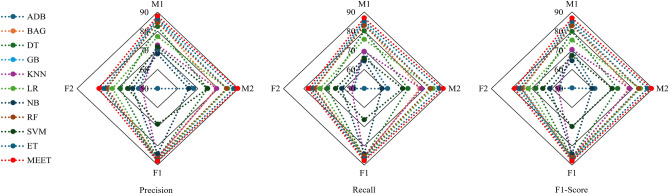
Performance metrics with classifier MEET.

The other performance metrics: Precision, Recall, and F1-score, are shown in Fig. 6. These findings are depicted as radar graphs; the lengths of the markers from the center indicate the strength of the performance metrics being displayed for a particular category. This graph enables visual comparison of several models and participants at once, with respect to a certain performance metric. Figure 6 examines the precision of the suggested model, which is an important metric in classification tasks. Precision measures the fraction of accurately predicted positive cases among all instances predicted positively by the model. This statistic is essential when the cost of false positives is high, since it directly influences the dependability and trustworthiness of the models’ predictions. From the graph analysis, it is evident that the proposed model’s Precision for subjects M1, M2, F1, and F2 is 87.90%, 89.70%, 88.10%, and 79.00%, respectively. These percentages reflect the model’s ability to identify relevant instances within each subject group reliably. Similarly, the second highest performing model among other MLCs is ET, with a precision score of 86.60%, 87.80%, 87.89%, and 76.72% for subjects M1, M2, F1, and F2, respectively. Among all the models, the highest true positive predictions are achieved by the MEET model, followed by ET. The lowest performance is observed with the ADB model.

Subjects M1, M2, F1, and F2 have recall values of 86.80%, 89.20%, 87.90%, and 78.49%, illustrating the MEET model’s exceptional ability to identify and retrieve relevant occurrences within each topic area reliably. Additionally, the second-highest recall value achiever among all subjects is ET, which is the expert of the MEET model. The recall values of ET across the subjects M1, M2, F1, and F2 are 85.21%, 87.30%, 87.37% and 76.61%, respectively. This evaluation of recall measures demonstrates the model’s greater performance in collecting all relevant events, reducing the likelihood of false negatives. By achieving high recall scores across several subjects, the model demonstrates its ability to identify significant information properly, enhancing its utility and reliability in a wide range of real-world scenarios.

Furthermore, the F1-score indicates the harmonic mean of precision and recall, allowing for a full evaluation of the MEET model’s performance across subjects M1, M2, F1, and F2. These participants had F1-scores of 86.90%, 89.00%, 87.77%, and 77.77%, respectively. The second highest attains F1-score among all subjects is ET, and the F1-score values for subjects M1, M2, F1, and F2 are 85.20%, 87.10%, 86.99%, and 76.44%, respectively. These statistics combine accuracy and recall, providing a fair assessment of the model’s ability to categorize cases while successfully reducing both false positives and false negatives. By attaining high F1-scores, the model displays its capacity to provide strong and consistent performance, establishing itself as a useful asset in a variety of practical applications and decision-making processes.

### Evaluation with publicly dataset

**Fig. 7:**
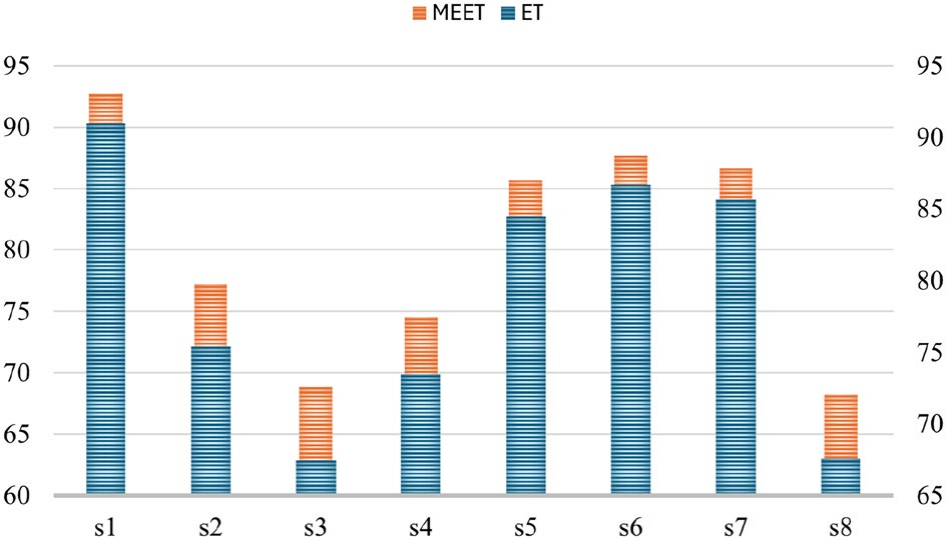
Accuracy analysis of the public dataset.

The robustness of the proposed architecture, MEET, was evaluated using the public data repository from R. N. Khushaba et al.^30,31^, which includes datasets of 15 different hand gesture classes. While performing the robustness analysis, the same steps are followed, and a similar environment is set up. After ensuring uniformity in the environment, the datasets are analyzed using the two best machine learning models: our proposed model, MEET, and the second most accurate model on the collected data, ET.

From these analyses, it was observed that the proposed architecture achieved the highest accuracy among all subjects (a total of eight). The accuracy attained by the subjects is shown in Fig. 7. The figure clearly shows that the accuracy achieved by subjects s1–s8 with the proposed MEET model had a mean increment in accuracy of approximately 1.25% for all subjects. Thus, in this setup, MEET attained the highest accuracy for both the collected and the publicly available datasets.

### Evaluation based on statistical analysis

**Table 3:** T-test statistics for data comparison.

Data	t-statistic	p-value
Acquired	5.7794	0.0103
Public	8.5526	0.0001

MEET-based results showed high accuracy, confirming the significance of the proposed framework. To validate statistical significance, a t-test was applied to the two datasets that were employed in this study. With the statistical parameters t-value and p-value, the t-test result is demonstrated in Table 3. The results signify that the p-value is less than 0.05 for both datasets. It shows that the proposed MEET framework consistently outperforms the simple classifier on both datasets.

### Ensuring model generalizability

Overfitting and bias are common issues encountered during classification^15,32,50^. To address these issues, MEET combines the knowledge of the Extra tree and the mixture of expert frameworks, which results in a generalized model.

- Learning with extra trees: the proposed model integrates an ensemble machine learning approach using Extra Trees, which is inherently resistant to overfitting. The Extra Trees model introduces randomness in both decision tree construction and feature subset selection, and it averages predictions across all random trees to control overfitting and improve generalization. By fitting multiple randomized decision trees on various data subsamples and averaging the predictions, the model effectively enhances predictive accuracy and minimizes overfitting.
- MoE framework: the proposed model is also inspired by the Mixture of Experts framework, where individual expert models are trained on specific classes of the datasets. Additionally, a gating mechanism is employed, trained on the entire dataset, which assigns weights to each expert to improve predictions. This structure reduces the risk of bias and further enhances the model’s generalizability, which has been explained in section Hand Gesture Recognition (HGR).

Following the discussion on model generalizability, further discussion on the computational efficiency of the MEET framework to support its applicability in real-time systems. The computational complexity during training of MEET relies on the number of expert classifiers and the employed gating model. Specifically, the complexity of the training can be approximated as: $\mathcal {O}(E.T.n.log n.d)$, where *E* represents the number of expert models, *T* represents the number of trees in each ET classifier, *n* is the number of training samples, and *d* indicates the maximum depth of the trees. Once the model is trained, the inference complexity of the MEET framework is significantly reduced to $\mathcal {O}(E.T.d)$ per input sample, as predictions are obtained through lightweight tree traversals followed by gated decision fusion. This low inference overhead makes MEET well suited for real-time hand gesture recognition tasks. Moreover, the ET supports efficient pruning and parallel execution, enabling decision-making with low latency. These properties make the proposed MEET framework computationally efficient and practically suitable for deployment on embedded and resource-constrained platforms commonly used in prosthetic control applications.

### Comparison with state-of-the-art studies

This study introduces a MEET framework for sEMG-based HGR. This framework helps to addressing overfitting and bias commonly observed in multi-class HGR problems. The effectiveness of the proposed framework has been validated by employing both an acquired dataset and a publicly available dataset. In the existing work in the literature mainly focuses on improving recognition of gesture by either increasing the number of channels, extracting a set of features, or employing advanced complex classifiers. However, many of these models rely on standalone machine learning models or single classifiers to trained on the complete dataset, which can lead to biased decision boundaries and reduced generalization. As summarized in Table 4, several studies employ DLCs or MLCs but lack a structured mechanism to mitigate class-level bias or explicitly combine specialized classifiers. On the other hand, the proposed MEET framework employs a divide-and-conquer strategy through the MoE paradigm, where multiple ET expert classifiers, which are trained on particular subsets of gesture, and a gating model integrate their outputs. This architectural design allows each expert to learn more discriminative boundaries for its assigned gestures, while the gating network reduces bias by adaptively weighting expert predictions. As shown in the table, although some studies report high accuracy, they often rely on higher channel counts, deeper models, or single classifiers without explicit bias-handling mechanisms. Another important advantage of MEET framework is built using tree-based models, decision-making processes are more transparent and easier to analyze compared to black-box deep learning models. This characteristic is particularly important for prosthetic and rehabilitation applications, where reliability and trustworthiness are critical.

**Table 4:** Detailed comparison with state-of-the-art studies.

REF	# CH	# HG	# FET	# MLC	Validated		SA	ACC (%)
					AQ	PUB		
^51^	12	17	4	4(3M+1D)	✗	✓	✓	85.9
^52^	-	27	9	4(3D+1M)	✗	✓	✗	85
^53^	6	7	10	4M	✓	✗	✓	97.18
^54^	6	10	-	1D	✓	✗	✓	92
This study	2	6	17	10M	✓	✓	✓	Mean: 85.57

*REF* reference, # *CH* no. of channel, # HG no. of hand gestures, # FET no. of features, # *MLC* no. of machine learning classifiers, *AQ* acquired dataset, *PUB* public dataset, *SA* statistical analysis, *ACC* accuracy, *M* machine learning, *D* deep learning.

## Conclusion and future works

This study presents an HGR framework, known as MEET, which integrates the strengths of Extra Trees and a mixture of expert frameworks to enhance the classification performance. The proposed framework is designed to address two common issues: overfitting and classification bias. Overfitting is addressed by an extra tree, which employs randomization in both feature selection and node splitting, as well as the aggregation of predictions across multiple trees and the use of bootstrapping. In parallel, bias is overcome by adopting the MoE Framework, where individual expert models are trained on a selected subset of the gesture classes rather than the full class datasets. Furthermore, a gating mechanism is utilized, which is trained on the entire dataset and assigns weights to each expert model to enhance the model predictions. This structure reduces the risk of bias, which enhances the model’s generalizability.

In this study, MEET is applied to identify hand gestures, and its performance is compared with that of ten other MLCs. Data is collected from four subjects—two adult males and two adult females—performing six distinct hand gestures, alongside a publicly available dataset containing 15 hand gestures, to perform the scalability analysis of the model. The results indicate that the MEET classifier outperformed the other MLCs, achieving a higher performance measure. Performance metrics, such as accuracy, precision, recall, and F1-score, for both the acquired dataset and the public dataset, indicated that MEET is an effective classifier for hand gesture identification.

This study primarily focuses on real-life HGR using sEMG signals. Gestures were classified using meaningful features extracted from the datasets, which include 17 features from both the time and frequency domains. Future work could involve optimal feature selection and assessing feature importance scores using explainable models within the framework of explainable artificial intelligence. Furthermore, the Extra Trees in the proposed framework currently employ default parameters, which could be further fine-tuned to enhance performance. This study employs sequential segmentation of the data for the proposed model for each expert. Future work could explore alternative data segmentation strategies for each expert. Furthermore, MEET could be combined with other machine learning techniques, such as deep learning, to create hybrid models that are even more effective.

Despite the promising results signifying the importance of the proposed MEET framework, the present work has several limitations. Such as the experimental evaluation, which is conducted on a limited number of subjects and considers only a predefined number of gestures. Although representative, it does not consider a wide range of hand movements. Future work will focus on validating the proposed framework on a larger and more diverse dataset, as well as inter-subject evaluation, and also potential muscle location variation. Additionally, future work will include participants with different physical health profiles, such as workouts or sports, etc., to make the framework more general and robust.

## Data Availability

The datasets used and/or analysed during the current study are available from the corresponding author on reasonable request.

## References

[1] Ozturk, H., Eraslan, B. & Gorur, K. Investigation of T-SNE and dynamic time warping within a unified framework for resting-state and minor analysis visual task-related EEG alpha frequency in biometric authentication: A detailed analysis. *Digit. Signal Process.* **160**, 105042 (2025).

[2] Gorur, K., Cetin, O., Ozer, Z. & Temurtas, F. Hospitalization status and gender recognition over the arboviral medical records using shallow and rnn-based deep models. *Results Eng.* **18**, 101109 (2023).

[3] Gorur, K., Olmez, E., Ozer, Z. & Cetin, O. EEG-driven biometric authentication for investigation of Fourier synchrosqueezed transform-ICA robust framework. *Arab. J. Sci. Eng.* **48**, 10901–10923 (2023).

[4] Wu, J., Li, X., Liu, W. & Wang, Z. J. SEMG signal processing methods: A review. *J. Phys. Conf. Ser.* **1237**, 032008 (IOP Publishing, 2019).

[5] Vijayvargiya, A., Singh, B., Kumar, R. & Tavares, J. M. R. Human lower limb activity recognition techniques, databases, challenges and its applications using SEMG signal: An overview. *Biomed. Eng. Lett.* **12**, 343–358 (2022). 36238368 10.1007/s13534-022-00236-w PMC9550908

[6] Montazerin, M. et al. Transformer-based hand gesture recognition from instantaneous to fused neural decomposition of high-density EMG signals. *Sci. Rep.* **13**, 11000 (2023). 37419881 10.1038/s41598-023-36490-w PMC10329032

[7] Kamavuako, E. N. On the applications of EMG sensors and signals (2022). 10.3390/s22207966 PMC9611382 36298317

[8] Reaz, M. B. I., Hussain, M. S. & Mohd-Yasin, F. Techniques of EMG signal analysis: Detection, processing, classification and applications. *Biol. Procedures Online* **8**, 11–35 (2006). 10.1251/bpo115 PMC1455479 16799694

[9] De Luca, C. J., Gilmore, L. D., Kuznetsov, M. & Roy, S. H. Filtering the surface EMG signal: Movement artifact and baseline noise contamination. *J. Biomech.* **43**, 1573–1579 (2010). 20206934 10.1016/j.jbiomech.2010.01.027

[10] Jlidi, N., Kouni, S., Jemai, O. & Bouchrika, T. Mediapipe with GNN for human activity recognition. *J. Univ. Comput. Sci. (JUCS)* **30** (2024).

[11] Asteris, P. G. et al. Genetic prediction of ICU hospitalization and mortality in COVID-19 patients using artificial neural networks. *J. Cell. Mol. Med.* **26**, 1445–1455 (2022). 35064759 10.1111/jcmm.17098 PMC8899198

[12] Gehlot, N., Soni, K., Kothari, P., Vijayvargiya, A. & Kumar, R. AI-enhanced diagnosis: Pediatric chest X-ray classification for bronchiolitis and pneumonia. In *2023 Seventh International Conference on Image Information Processing (ICIIP)*. 753–758 (IEEE, 2023).

[13] Gill, H. S. & Khehra, B. S. An integrated approach using cnn-rnn-lstm for classification of fruit images. *Mater. Today Proc.* **51**, 591–595 (2022).

[14] Jlidi, N., Kouni, S., Jemai, O. & Bouchrika, T. Graph-based joint detection and tracking with Euclidean edges for multi-object video analysis. *Displays* 103229 (2025).

[15] Asteris, P. G., Skentou, A. D., Bardhan, A., Samui, P. & Pilakoutas, K. Predicting concrete compressive strength using hybrid ensembling of surrogate machine learning models. *Cement Concr. Res.* **145**, 106449 (2021).

[16] Atzori, M., Cognolato, M. & Müller, H. Deep learning with convolutional neural networks applied to electromyography data: A resource for the classification of movements for prosthetic hands. *Front. Neurorobot.* **10**, 9 (2016). 27656140 10.3389/fnbot.2016.00009 PMC5013051

[17] Jlidi, N., Jemai, O. & Bouchrika, T. Enhancing human action recognition through transfer learning and body articulation analysis. *Circuits Syst. Signal Process.* **44**, 4394–4422 (2025).

[18] Côté-Allard, U. et al. Deep learning for electromyographic hand gesture signal classification using transfer learning. *IEEE Trans. Neural Syst. Rehabil. Eng.* **27**, 760–771 (2019). 30714928 10.1109/TNSRE.2019.2896269

[19] Geng, W. et al. Gesture recognition by instantaneous surface EMG images. *Sci. Rep.* **6**, 36571 (2016). 27845347 10.1038/srep36571 PMC5109222

[20] Huang, D. & Chen, B. Surface EMG decoding for hand gestures based on spectrogram and cnn-lstm. In *2019 2nd China Symposium on Cognitive Computing and Hybrid Intelligence (CCHI)*. 123–126 (IEEE, 2019).

[21] Toro-Ossaba, A. et al. Lstm recurrent neural network for hand gesture recognition using EMG signals. *Appl. Sci.* **12**, 9700 (2022).

[22] Jena, A., Sharma, P., Gehlot, N., Vijayvargiya, A. & Kumar, R. Efficient contaminant identification in SEMG signals using machine learning. In *2024 Third International Conference on Power, Control and Computing Technologies (ICPC2T)*. 25–30 (IEEE, 2024).

[23] Toledo-Pérez, D. C., Rodríguez-Reséndiz, J., Gómez-Loenzo, R. A. & Jauregui-Correa, J. Support vector machine-based EMG signal classification techniques: A review. *Appl. Sci.* **9**, 4402 (2019).

[24] Abbaspour, S., Lindén, M., Gholamhosseini, H., Naber, A. & Ortiz-Catalan, M. Evaluation of surface EMG-based recognition algorithms for decoding hand movements. *Med. Biol. Eng. Comput.* **58**, 83–100 (2020). 31754982 10.1007/s11517-019-02073-z PMC6946760

[25] Bhagwat, S. & Mukherji, P. Electromyogram (EMG) based fingers movement recognition using sparse filtering of wavelet packet coefficients. *Sādhanā* **45**, 1–11 (2020).

[26] Lee, J.-W. & Yu, K.-H. Wearable drone controller: Machine learning-based hand gesture recognition and vibrotactile feedback. *Sensors* **23**, 2666 (2023). 36904870 10.3390/s23052666 PMC10006975

[27] Fatimah, B., Singh, P., Singhal, A. & Pachori, R. B. Hand movement recognition from SEMG signals using Fourier decomposition method. *Biocybern. Biomed. Eng.* **41**, 690–703 (2021).

[28] Jacobs, R. A., Jordan, M. I., Nowlan, S. J. & Hinton, G. E. Adaptive mixtures of local experts. *Neural Comput.* **3**, 79–87 (1991). 31141872 10.1162/neco.1991.3.1.79

[29] Jordan, M. I. & Jacobs, R. A. Hierarchical mixtures of experts and the EM algorithm. *Neural Comput.* **6**, 181–214 (1994).

[30] Khushaba, R. N. & Kodagoda, S. Electromyogram (EMG) feature reduction using mutual components analysis for multifunction prosthetic fingers control. In *2012 12th International Conference on Control Automation Robotics & Vision (ICARCV)*. 1534–1539 (IEEE, 2012).

[31] Khushaba, R. N. Individual and combined fingers movements. https://rami-khushaba.com/biosignals-repository.

[32] Gehlot, N., Jena, A., Vijayvargiya, A. & Kumar, R. Surface electromyography based explainable artificial intelligence fusion framework for feature selection of hand gesture recognition. *Eng. Appl. Artif. Intell.* **137**, 109119 (2024).

[33] Gehlot, N. et al. L-shade optimized learning framework for SEMG hand gesture recognition. *Sci. Rep.* **15**, 36562 (2025). 41120376 10.1038/s41598-025-20076-9 PMC12540717

[34] Shi, W.-T., Lyu, Z.-J., Tang, S.-T., Chia, T.-L. & Yang, C.-Y. A bionic hand controlled by hand gesture recognition based on surface EMG signals: A preliminary study. *Biocybern. Biomed. Eng.* **38**, 126–135 (2018).

[35] Hamrick, M. W., Churchill, S. E., Schmitt, D. & Hylander, W. L. EMG of the human flexor pollicis longus muscle: Implications for the evolution of hominid tool use. *J. Hum. Evolut.* **34**, 123–136 (1998). 10.1006/jhev.1997.0177 9503091

[36] Su, K. L. *Analog Filters* (Springer, 2012).

[37] Vijayvargiya, A., Gupta, V., Kumar, R., Dey, N. & Tavares, J. M. R. A hybrid WD-EEMD SEMG feature extraction technique for lower limb activity recognition. *IEEE Sens. J.* **21**, 20431–20439 (2021).

[38] Gehlot, N., Jena, A., Vijayvargiya, A. & Kumar, R. SEMG-based classification of finger movement with machine learning. In *2023 International Conference on Computer, Electronics & Electrical Engineering & their Applications (IC2E3)*. 1–6 (IEEE, 2023).

[39] Quinlan, J. R. Improved use of continuous attributes in c4. 5. *J. Artif. Intell. Res.* **4**, 77–90 (1996).

[40] Indra, S., Wikarsa, L. & Turang, R. Using logistic regression method to classify tweets into the selected topics. In *2016 International Conference on Advanced Computer Science and Information Systems (ICACSIS)*. 385–390 (IEEE, 2016).

[41] Breiman, L. Random forests. *Mach. Learn.* **45**, 5–32 (2001).

[42] Friedman, J. H. Greedy function approximation: A gradient boosting machine. *Ann. Stat.* 1189–1232 (2001).

[43] Dudani, S. A. The distance-weighted k-nearest-neighbor rule. In *IEEE Transactions on Systems, Man, and Cybernetics*. 325–327 (1976).

[44] Geurts, P., Ernst, D. & Wehenkel, L. Extremely randomized trees. *Mach. Learn.* **63**, 3–42 (2006).

[45] Freund, Y. & Schapire, R. E. A decision-theoretic generalization of on-line learning and an application to boosting. *J. Comput. Syst. Sci.* **55**, 119–139 (1997).

[46] Cristianini, N. & Shawe-Taylor, J. *An Introduction to Support Vector Machines and Other Kernel-Based Learning Methods* (Cambridge University Press, 2000).

[47] Breiman, L. Bagging predictors. *Mach. Learn.* **24**, 123–140 (1996).

[48] Gehlot, N., Malik, S., Jena, A., Vijayvargiya, A. & Kumar, R. Xai-driven SEMG feature analysis for hand gestures. In *2024 Third International Conference on Power, Control and Computing Technologies (ICPC2T)*. 19–24 (IEEE, 2024).

[49] Pedregosa, F. et al. Scikit-learn: Machine learning in python. *J. Mach. Learn. Res.* **12**, 2825–2830 (2011).

[50] Apostolopoulou, M. et al. Mapping and holistic design of natural hydraulic lime mortars. *Cement Concr. Res.* **136**, 106167 (2020).

[51] Prakash, K. S. & Kunju, N. An optimized electrode configuration for wrist wearable EMG-based hand gesture recognition using machine learning. *Expert Syst. Appl.* **274**, 127040 (2025).

[52] Sakinala, U. C. & Abinaya, S. Enhanced detection of hand gestures from SEMG signals using stacking ensemble with particle swarm optimization and meta-classifier. (IEEE Access, 2025).

[53] Xiong, D. et al. Robotic telemanipulation with EMG-driven strategy-assisted shared control method. *Sci. China Technol. Sci.* **67**, 3812–3824 (2024).

[54] Asif, A. R. et al. Performance evaluation of convolutional neural network for hand gesture recognition using EMG. *Sensors* **20**, 1642 (2020). 32183473 10.3390/s20061642 PMC7146563

